# Correlation of Cytokine Release Syndrome With Prognosis After Chimeric Antigen Receptor T Cell Therapy: Analysis of 54 Patients With Relapsed or Refractory Multiple Myeloma

**DOI:** 10.3389/fimmu.2022.814548

**Published:** 2022-04-27

**Authors:** Xue Wang, Lina Zhao, Jing Wang, Yue Yao, Jiaojiao Wang, Shengwei Ji, Tian Hua, Shiyuan Wang, Hai Cheng, Ming Shi, Zhenyu Li, Lingyu Zeng, Junnian Zheng, Kailin Xu, Jiang Cao

**Affiliations:** ^1^Department of Hematology, The Affiliated Hospital of Xuzhou Medical University, Xuzhou, China; ^2^The First Clinical Medical College, Xuzhou Medical University, Xuzhou, China; ^3^Cancer Institute, Xuzhou Medical University, Xuzhou, China; ^4^Jiangsu Bone Marrow Stem Cell Institute, Xuzhou, China

**Keywords:** multiple myeloma, cytokine release syndrome, chimeric antigen receptor T cell, CD19, B cell maturation antigen

## Abstract

Although chimeric antigen receptor T (CAR-T) cell therapy has proven to be effective in treating relapsed or refractory multiple myeloma (R/R MM), the severity of cytokine release syndrome (CRS) can affect patient survival and the risk factors for CRS remain an intractable issue. We enrolled 54 patients with R/R MM following combined infusion of anti-CD19 and anti-B-cell maturation antigen (BCMA) CAR-T cells. The results showed the overall response rate was 94% (51/54) after CAR-T cell infusion, with a 100% incidence of CRS, including 47 patients with grade 1-2 (mild) CRS and 7 patients with grade 3-5 (severe) CRS. In the mild CRS group, the median progression-free survival (PFS) was 18.2 months (95% CI, 6.5 to 30.1) and the median overall survival (OS) was not reached yet. In the severe CRS group, median PFS and median OS were 1.9 months (95% CI, 0.2 to 3.8). Further analysis demonstrated that severe CRS had a shorter median PFS and OS than mild CRS (*p*=0.029, *p*=0.020). Bone marrow tumor burden was found to be independently associated with CRS. The grade of CRS was positively correlated with six serum cytokines levels including G-CSF, IL-6, IL-8, IP-10, MIP-1a and RANTES. In conclusion, early detection and management of CRS are imperative for the prevention of life-threatening complications and improvement in the survival of patients of CAR-T cell therapy.

**Clinical Trial Registration:**

www.chictr.org.cn, identifier ChiCTR-OIC-17011272.

## 1 Introduction

Chimeric antigen receptor T (CAR-T) cells are engineered molecules that combine the recognition ability of specific tumor antigens with the powerful anti-tumor effects of T cells. CAR-T cell therapy is emerging as a promising therapeutic option for treating relapsed/refractory multiple myeloma(RR MM). Multiple studies have shown that the anti-B-Cell maturation antigen (BCMA) or combination of anti-CD19 and anti-BCMA CAR-T cells has an encouraging activity in the treatment of R/R MM, with an overall response rate of 70-100% (1–4). However, various adverse effects such as cytokine release syndrome (CRS), immune effector cell-associated neurotoxicity syndrome (ICANS), and cytopenias constitute preventing the widespread application of CAR-T cell therapy (4). CRS resulting from rapid immune activation induced by CAR-T cells is the most significant treatment related toxicity. CRS initially manifests with fever and can progress to life-threatening capillary leak with hypoxia and hypotension. However, whether patients with severe CRS affect the response or long-term prognosis post CAR-T therapy remains unclear. With the increasing role of CAR-T therapy, understanding the unique complications of this therapy and their effects on long-term outcomes is increasingly important. Our previous analysis confirm that the combined infusion of humanized anti-CD19 and anti-BCMA CAR-T cells is feasible and the majority of patients with R/R MM achieved a response (4). Here, we further expanded the sample size and systematically analyzed the correlation between CRS and prognosis. Notably, we aimed to dissect risk factors among patients with R/R MM participating in phase 1 clinical trial of CAR-T cell therapy.

## 2 Methods

### 2.1 Patient Selection

A total of 54 patients with R/R MM who underwent CAR-T cell therapy between July 2017 to August 2020 were retrospectively reviewed (ChiCTR-OIC-17011272). All patients were enrolled in phase 1 open-label single-center clinical trials of CAR-T cell therapy targeting BCMA and CD19 and met the International Myeloma Working Group (IMWG) diagnostic criteria for R/R MM, which is defined as a disease that progresses on salvage therapy or progresses within 60 days of the last treatment in patients who previously achieved at least a minimal response to treatment (5). Female patients had to be human chorionic gonadotropin-negative, with no plans for pregnancy within 6 months of treatment. Patients with mental or psychological illnesses, severe allergies, or a history of severe allergies (especially those who were allergic to interleukin [IL]-2) were excluded.

### 2.2 CAR-T Cell Manufacture and Clinical Protocol

Peripheral blood mononuclear cells were collected from enrolled patients for CAR-T cell generation. The protocol for CAR-T cell manufacture in our center has been described previously (6). Patients received conditioning chemotherapy consisting of cyclophosphamide (750mg per square meter of body-surface, day -5) and fludarabine (30mg per square meter per day, days -5, -4, and -3). Anti-BCMA CAR-T cells and humanized anti-CD19 CAR-T cells were sequentially infused at a dose of 1×10^6^ cells per kilogram of bodyweight, respectively, on day 0. The vital signs were recorded at any time during treatment. Tocilizumab was used to management CRS in patients with grade ≥2 CRS. The serum levels of 23 cytokines were collected daily from the onset of CRS to the end of CRS and detected by Cytometric Bead Array (CBA) including IL-2, IL-3, IL-4, IL-5, IL-6, IL-7, IL-8, IL-9, IL-10, IL-1β, IL-12P70, colony stimulating factor (G-CSF, GM-CSF), interferon (γ-interferon), tumor necrosis factor (TNF), growth factor (VEGF, b-FGF), chemokine (MIP-1a, MIG, IP-10, Eotaxin, RANTES, MCP-1).

### 2.3 Overall Response and CRS

Responses were assessed by updated International Myeloma Working Group criteria (7), including stringent complete response (sCR), complete response (CR), very good partial response (VGPR), partial response (PR), minimal response (MR), stable disease (SD) and progressive disease (PD). Minimal residual disease (MRD) was evaluated according to EuroFlow protocol (8) using bone marrow aspirates, and MRD-negativity was defined as the absence of phenotypically aberrant clonal plasma cells with a minimum sensitivity of 10^-5^ monoclonal myeloma cells among mononuclear cells. CRS effects were graded and managed according to the recommendations of Lee et al. (9). Grade 1-2 CRS was “mild”, while grade 3-5 CRS was “severe.” Other toxicities were assessed according to the National Cancer Institute’s Common Terminology Criteria for Adverse Events, version 5.0 (CTCAE 5.0) (10). Overall response and CRS were assessed by three experienced clinicians.

### 2.4 Statistical Analysis

The time of data cutoff for the current analysis was May 31, 2021. Descriptive statistics included medians with minimum and maximum for continuous variables and counts and percentages for categorical variables. Two-sided 95% exact confidence intervals (CIs) based on the exact method of binomial distribution were calculated for each response category. Spearman correlations were used to measure the correlations between two continuous variables. The continuous variables were tested by Mann-Whitney U test for two groups. Univariate and multivariable logistic regression was used to estimate the risk factors of the severity of CRS. Log-rank test was used to estimate the duration of response, progression-free survival (PFS) and overall survival (OS). A p-value <0.05 was considered significant. Analysis was performed using GraphPad Prism version 6.0.

## 3 Results

### 3.1 Patient Characteristics

From July 1, 2017 to August 31, 2020, 54 patients were enrolled and treated with a combination of anti-BCMA and humanized anti-CD19 CAR-T cells. The median follow-up of patients was 19.6 months (range, 0.6 to 48.1) after CAR-T cells infusion. Patient characteristics were shown in **Table 1**. The median age was 58 years (range, 30 to 67), and the median time from diagnosis to CAR-T cells infusion was 30 months (range, 8 to 167). 24 patients (44%) had International Staging System stage III disease and 13 patients (24%) had extramedullary disease. We defined the plasma cell count greater than 50% on bone marrow biopsy as a high tumor burden. So 13 patients (24%) have a high tumor burden. Before the lymphodepletion regimen, patients received a median of four chemotherapy cycles (range, 2 to 17) and 15 patients (28%) received autologous hematopoietic stem cell transplantation (HSCT). Fifty-two (96%) patients received both proteasome inhibitor and immunomodulatory drug previously, 15 (28%) were exposed to ixazomib, 3 (6%) pomalidomide, and 7 (13%) daratumumab. Two patients (3%) proceeded to autologous HSCT and the other patients received no further consolidation therapy post CAR-T cells infusion.

**Table 1:** Demographics and baseline disease characteristics.

Characteristics	(N=54)
Age, no. (%)	
<65 yr	46 (85)
≥65 yr	8 (15)
Mean (SD)	57 (8)
Median (range)	58 (53 to 62)
Sex, no. (%)	
Male	28 (52)
Female	26 (48)
ISS stage, no. (%)	
I	7 (13)
II	23 (43)
III	24 (44)
Bone marrow monoclonal plasma cell ratio (%)	
<50%	41 (76)
≥50%	13 (24)
Type of myeloma, no. (%)	
IgG	27 (50)
IgA	7 (13)
IgD	4 (7)
IgM	1 (2)
Light chain	11 (20)
Kappa	7 (13)
Lambda	4 (7)
Nonsecretory	4 (7)
Time from initial MM diagnosis, months	
Mean (SD)	40 (30)
Median (range)	30 (18 to 61)
Number of prior lines of therapy, no	
Mean (SD)	4 (2)
Median (range)	4 (3 to 5)
Autologous stem cell transplantation, no. (%)	15 (28)
Prior therapies, no. (%)	
Proteasome inhibitors	53 (98)
Bortezomib	52 (96)
Ixazomib	15 (28)
Immunomodulatory agents	53 (98)
Lenalidomide	34 (63)
Pomalidomide	3 (6)
Thalidomide	38 (70)
Proteasome inhibitors+immunomodulatory agents	52 (96)

### 3.2 Response Rates

**Figure 1:**
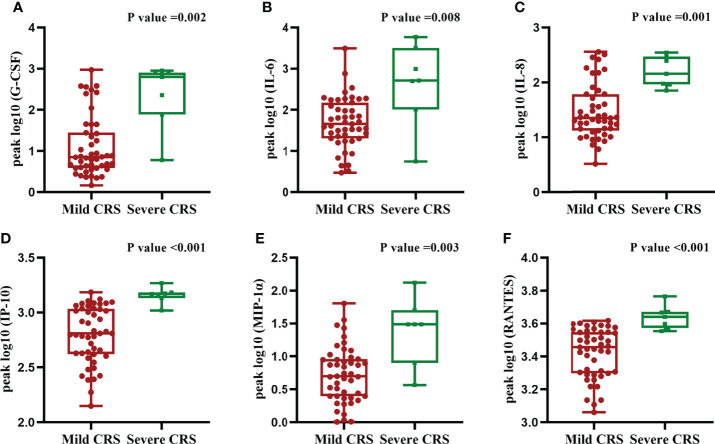
CRS grade correlate with serum cytokine. **(A–F)** Grade of CRS and the levels of G-CSF, IL-6, IL-8, IP-10, MIP-1a and RANTES. Two-sided P-values calculated based on Spearman correlations.

The overall response (PR or better) within three months to CAR-T cell therapy was 94% (51/54), including 17 sCR, 12 CR, 12 VGPR, and 10 PR. MRD status was negative for 40/54 (83%) patients. Based on two-logistic regression analysis, the response was insignificantly associated with severity of CRS (P>0.05) **(** **Table 2**). Based on Kaplan-Meier estimates, the median duration of response was 18.2 months (95% CI, 8.3 to 28.1). The median PFS for these 54 patients was 16.9 months (95% CI, 8.5 to 25.3) (**Table 2**), with PFS rates of 53% (95% CI, 42 to 68) at 12 months and 39% (95% CI, 26 to 52) at 24 months. The median OS for 54 patients was not obtained yet (**Table 2**), with OS rates of 75% (95% CI, 63 to 84) at 12 months, and 68% (95%CI, 54 to 79) at 24 months (**Figure 1**). At data cutoff, 20 patients (37%) remained progression free with a median of 21.3 month (95% CI, 19.3 to 28.8) and 18 patients (33%) died during the follow-up.

**Table 2:** Severity of CRS with response post CAR-T cells infusion.

Response Category	Severity of CRS		
	Mild CRS (N=47)	Severe CRS (N=7)	P
Overall response			
No. with response	44	7	
Rate — % (95% CI)	94 (86-100)	100 (100-100)	1.000
Best overall response — no. (%)			
Complete response or better	24 (51)	5 (71)	0.431
Complete response	11 (23)	1 (14)	
Stringent complete response	13 (28)	4 (57)	
Very good partial response or better	35 (74)	6 (86)	1.000
Very good partial response	11 (23)	1 (14)	
Partial response	9 (19)	1 (14)	
Stable disease	3 (6)	0	
Progressive disease	0	0	

### 3.3 CRS and Neurotoxicity

After CAR-T cells infusion, 100% (54/54) of the patients developed CRS. 47 patients (87%) sustained mild CRS and 7 patients (13%) developed severe CRS, respectively. In the mild CRS group, median time to CRS onset was 7 days (range 0-13) with a median duration of 4 days (range 1-9); 10 of 47 patients received tocilizumab and 18 of 47 patients received glucocorticoids. In the severe CRS group, the median time to CRS onset was 2 days (range 0-5) with a median duration of 7 days (range 0-36); 6 of 7 patients received tocilizumab and 7 of 7 patients received glucocorticoids. 3 of 7 patients with severe CRS required vasopressor support for hypotension, and one patient required mechanical ventilation One patient died from grade 5 CRS. In addition, 2 of 54 patients (4%) respectively developed grade 3 and grade 4 neurotoxicity with recurrent seizures and mild cerebral edema on magnetic resonance imaging (MRI) that were fully resolved after treatment with high-dose methylprednisolone (1 g/day x 3). Moreover, patients with neurotoxicity had a high tumor burden, and one with grade 3 CRS and another with grade 4 CRS. Based on Kaplan-Meier estimates, in the mild CRS group, the median PFS was 18.2 months (95% CI, 6.5 to 30.1); median OS was not reached yet. In the severe CRS group, the median PFS and median OS were 1.9 months (95% CI, 0.2 to 3.8). Further analysis demonstrated that severe CRS had a shorter median PFS and OS than mild CRS (*p*=0.029, *p*=0.020). Multivariate Cox regression analysis was not performed for PFS or OS due to the small number of patients with severe CRS (**Figure 1**).

### 3.4 Factors Associated With CRS

#### 3.4.1 Serum Cytokine Correlate With CRS

**Figure 2:**
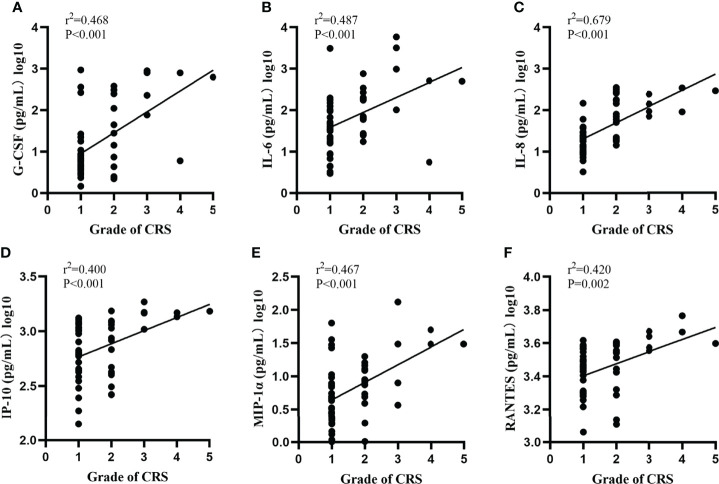
Severity of CRS correlate with the peak levels of serum cytokine. **(A–F)** Severity of CRS and the peak levels of G-CSF, IL-6, IL-8, IP-10, MIP-1a and RANTES. Two-sided P-values calculated based on Mann-Whitney U test.

**Figure 3:**
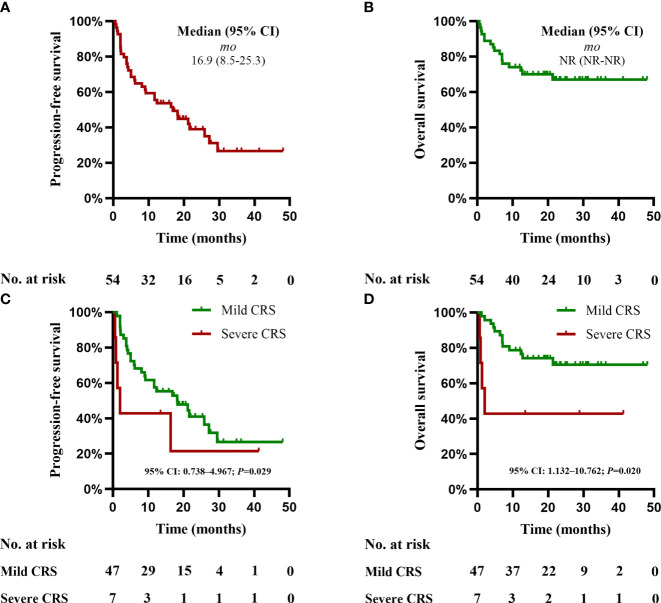
Progression-free survival (PFS) and overall survival (OS). **(A)** The PFS in all the 54 patients. **(B)** The OS in all the 54 patients. **(C)** The PFS according to severity of CRS. **(D)** The OS according to severity of CRS. Two-sided P-values calculated based on Kaplan-Meier estimates.

We quantified 23 cytokines in peripheral blood serum after CAR-T cells infusion and found that the grade of CRS was positively correlated with six serum cytokines levels including G-CSF, IL-6, IL-8, IP-10, MIP-1a and RANTES (r^2 ^= 0.468, *p*<0.001; r^2 ^= 0.487, *p*<0.001; r^2 ^= 0.679, *p*<0.001; r^2 ^= 0.400, *p*<0.001; r^2 ^= 0.467, *p*<0.001, r^2 ^= 0.420, *p*=0.002) (**Figure 2**). We did not observe significant differences in other cytokines. Moreover, the peak levels of G-CSF (*p*=0.002), IL-6(*p*=0.008), IL-8(*p*=0.001), IP-10(*p*<0.001), MIP-1a (*p*=0.003)and RANTES(*p*<0.001)were significantly higher in patients with severe CRS compared to those with mild CRS (**Figure 3****)**.

#### 3.4.2 Patients’ Characteristics and CRS

Based on univariate logistic regression analysis, the severity of CRS was not associated with patients’ characteristics including age, sex, disease type, disease stage, extramedullary disease, number of previous therapy lines, the time since diagnosis (*p>*0.05). In addition, patients with severe CRS had higher levels of bone marrow plasma cells compared with patients with mild CRS, which indicates an increased chance of severe CRS in patients with high bone marrow tumor burdens (OR: 1.034; 95% CI: 1.001-1.069; *p*=0.045). Based on univariate analysis, age and disease burden were entered in an ordinal logistic regression model,bone marrow tumor burden was found to be independently associated with CRS (**Table 3**). This finding is consistent with a previous study (11). Additionally, we found that administration of tocilizumab was correlated with severe CRS (OR: 22.200; 95% CI: 2.389-206.308; *p*=0.006). Univariate analyses showed that earlier to CRS onset was positively correlated with severe CRS (OR: 0.686; 95% CI: 0.514-0.916; *p*=0.011), and a longer duration of CRS was also positively correlated with severe CRS (OR: 3.445; 95% CI: 1.471-8.068; *p*=0.004).

**Table 3:** Factors associated with CRS severity post CAR-T cells infusion on univariable and multivariable analysis.

Variable	Mild CRS	Severe CRS	Univariate Analysis		Multivariable Analysis	
			OR	*P*	OR	*P*
N (%)	47 (87)	7 (13)				
Age (range)	59 (53 to 63)	56 (45 to 57)	0.147	0.086	0.899	0.902
Male sex — no. (%)	24 (51)	4 (57)	0.783	0.764		
Type of myeloma				0.789		
Extramedullary disease, *n* (%)	14 (30)	1 (14)	0.393	0.407		
Number of prior lines of therapy (range)	4 (3 to 5)	4 (3 to 5)	0.896	0.620		
Time from initial MM diagnosis (range)	30 (18 to 51)	32 (15 to 59)	0.996	0.759		
Bone marrow tumor burden (range)	20 (5 to 32)	47 (34 to 56)	1.034	0.045	1.046	0.046

## 4 Discussion

Given that CRS is potentially life threatening side effect during the management of patients with R/R MM receiving CAR-T cell therapy, the major focus of clinical trials is to identify risk factors that are potentially modifiable (12–14). However, a little proportion of the literature has focused on the correlation between CRS and patient prognosis post-CAR-T. Here, to address this, we analyzed the correlation between CRS and prognosis. Meanwhile, we aimed to dissect risk factors of CRS following CAR-T cells infusion, in a large cohort of patients with R/R MM.

In this retrospective analysis, an overall response rate of 94% and an MRD-negative rate of 83% were observed. Meanwhile, we also found that 100% of the patients developed CRS after CAR-T cells infusion. The incidence of severe CRS was 13% in our study which is consistent with the results reported in previous studies. Mailankody et al. (15) found that the incidence of severe CRS was 20% in the phase I dose escalation clinical trial including 11 R/R MM patients who received BCMA CAR- T cells infusions. While Cohen AD et al. (2) found that the incidence of severe CRS was 32% in 25 MM patients post BCMA CAR-T cells infusion. Xu J et al. (1) also found that the incidence of severe CRS was 41% in 17 R/R MM patients who received BCMA CAR- T cells infusions. We considered that the reason for the relatively low incidence of severe CRS in our study may be related to the active management such as timely usage of tocilizumab and lower dose CAR-T cells transfusions.

To our knowledge, our results provide the longest follow-up of patients with R/R MM treated with the combination of anti-BCMA and humanized anti-CD19 CAR T-cells. Currently, most clinical research focuses on the risk factors of the long-term prognosis after CAR-T cell therapy in MM patients. However, whether the severity of CRS has an impact on long-term survival is rarely reported. In our study, we did identify a difference in PFS and OS between patients with mild CRS and severe CRS. Our results showed that severe CRS in myeloma patients is associated with poorer clinical outcomes although the exact mechanism is not clear. Therefore, clarifying the risk factors for CRS and searching for early warning indicators for severe CRS will help clinicians to diagnose and intervene in time.

Currently, the pathophysiology of CRS is not completely understood, but it is commonly considered to occur due to the on-target effects of CAR-T cells and somehow triggers activation of bystander cells (both immune cells and nonimmune cells) in the tumor environment (16). Activation of these bystander cells initiates the proinflammatory process and the massive production of cytokines, which eventually causes CRS. In this trial, the univariate analysis highlighted the bone marrow tumor burden, time to CRS onset, and duration as risk factors for severe CRS, which is consistent with the findings previously reported (12, 17).

Previous studies confirmed that in some hematological diseases, such as acute lymphoblastic leukemia and lymphoma after CAR-T cells therapy, patients with severe CRS presented elevated levels of specific cytokines compared with those who did not develop severe CRS (9, 18). Notable cytokines differentially elevated in severe CRS included IFN-γ, CRP, ferritin, IL-6, IL-8, G-CSF, IP-10 and MIP-1a (12, 19, 20). In this study, similar to previous studies, we not only found that plasma levels of IL-6, IL-8, G-CSF, IP-10 and MIP-1a were positively correlated with the grade of CRS, but also confirmed that the peripheral blood peak concentrations of these five factors were positively correlated with the severity of CRS.

RANTES, also known as CC chemokine ligand 5 (CCL5), a proinflammatory CC-chemokine, is a member of the chemokine family that regulates cell migration, which can be secreted by activated T lymphocytes and mononuclear macrophages. Our results showed that the plasma level of RANTES was positively correlated with the severity of CRS, which has not been reported in previous studies. We consider the rise of RANTES for the following reasons. First, previous studies have shown that CRS was associated with activation of T lymphocytes and mononuclear macrophages (21, 22). So severe CRS might stimulate the release of a large amount of RANTES into the blood. Second, RANTES mediates the migration and navigation of classical lymphoid cells like T cells, monocytes, basophils, eosinophils, natural killer cells, dendritic and mast cells. The interaction between these cells can further promote the secretion of a variety of inflammatory cytokines, thus aggravating the severity of CRS. Third, studies have shown that when the body is stimulated by inflammatory cytokines, the function of endothelial cells of large vessels is active, and the T lymphocytes and mononuclear macrophages attached to its surface secrete more RANTES, and the RANTES receptor is in a high expression state. Our results suggest that dynamic measurement of the concentration of multiple cytokines and inflammatory factors may be beneficial for the timely diagnosis and actively treat severe CRS.

In conclusion, based on relatively large sample size and longer follow-up time, our results showed that the combination of anti-BCMA and humanized anti-CD19 CAR-T cells induced durable response in R/R MM patients with a safe long-term profile. Severe CRS was negatively associated with long-term prognosis in patients and multiple factors were associated with the severity of CRS. Therefore, recognition of associated risk factors for severe CRS is critical for the incidence and mortality of severe CRS. Meanwhile, early detection and management of CRS are imperative for the prevention of life-threatening complications and improvement in the survival of patients of these innovative therapies.

## Data Availability Statement

The original contributions presented in the study are included in the article. Further inquiries can be directed to the corresponding authors.

## Ethics Statement

The studies involving human participants were reviewed and approved by the Ethics Committee of the Affiliated Hospital of Xuzhou Medical University. The patients/participants provided their written informed consent to participate in this study.

## Author Contributions

XW, LZ, JW, YY, JJW, SJ, TH, SW, HC, and MS conducted experiments. ZL, LZ, JZ, KX, and JC conceived experimental designs and analyzed experimental data. XW, LYZ and JW wrote the manuscript. All authors contributed to the article and approved the submitted version.

## Funding

This work was supported by the grants from the Natural Science Foundation of China (81930005), Key Research & Development Plan of Jiangsu Province (BE2018634), Xuzhou Medical Leading Talents Training Program (XWRCHT20210028), and Key Research & Development Plan of Xuzhou (KC18102).

## Conflict of Interest

The authors declare that the research was conducted in the absence of any commercial or financial relationships that could be construed as a potential conflict of interest.

## Publisher’s Note

All claims expressed in this article are solely those of the authors and do not necessarily represent those of their affiliated organizations, or those of the publisher, the editors and the reviewers. Any product that may be evaluated in this article, or claim that may be made by its manufacturer, is not guaranteed or endorsed by the publisher.
